# P-416. From Broad to Best: A Structured, Automated, and Scalable EHR Approach to Evaluate Empiric Antibiotic Appropriateness

**DOI:** 10.1093/ofid/ofaf695.633

**Published:** 2026-01-11

**Authors:** Wenyuan Chen, Nicholas P Marshall, Fatemeh Amrollahi, Fateme Nateghi Haredasht, Manoj Maddali, Stephen Ma, Amy Chang, Stan Deresinski, Mary Kane Goldstein, Steven Asch, Niaz Banaei, Hayden T Schwenk, Jonathan H Chen

**Affiliations:** Stanford University, Palo Alto, California; Stanford University, Palo Alto, California; Stanford University, Palo Alto, California; Stanford University, Palo Alto, California; Stanford University, Palo Alto, California; Stanford, Palo Alto, California; Stanford University, Palo Alto, California; Stanford Health Care, Stanford, CA; Stanford University, Palo Alto, California; Stanford University, Palo Alto, California; Stanford University School of Medicine, Palo Alto, CA; Stanford University School of Medicine, Palo Alto, CA; Stanford University, Palo Alto, California

## Abstract

**Background:**

Current antimicrobial stewardship metrics emphasize reducing overall antibiotic use but rarely assess patient-level appropriateness. Tools like the SAAR and EHR alerts benchmark use or trigger rules but do not evaluate whether an agent was clinically appropriate. We developed an automated, scalable metric using SQL, widely adopted and optimized for querying data, to implement the DOOR MAT (Desirability of Outcome Ranking for the Management of Antimicrobial Therapy) framework, which ranks empiric antibiotics by spectrum, favoring narrower agents when susceptible.

**Figure 1:**
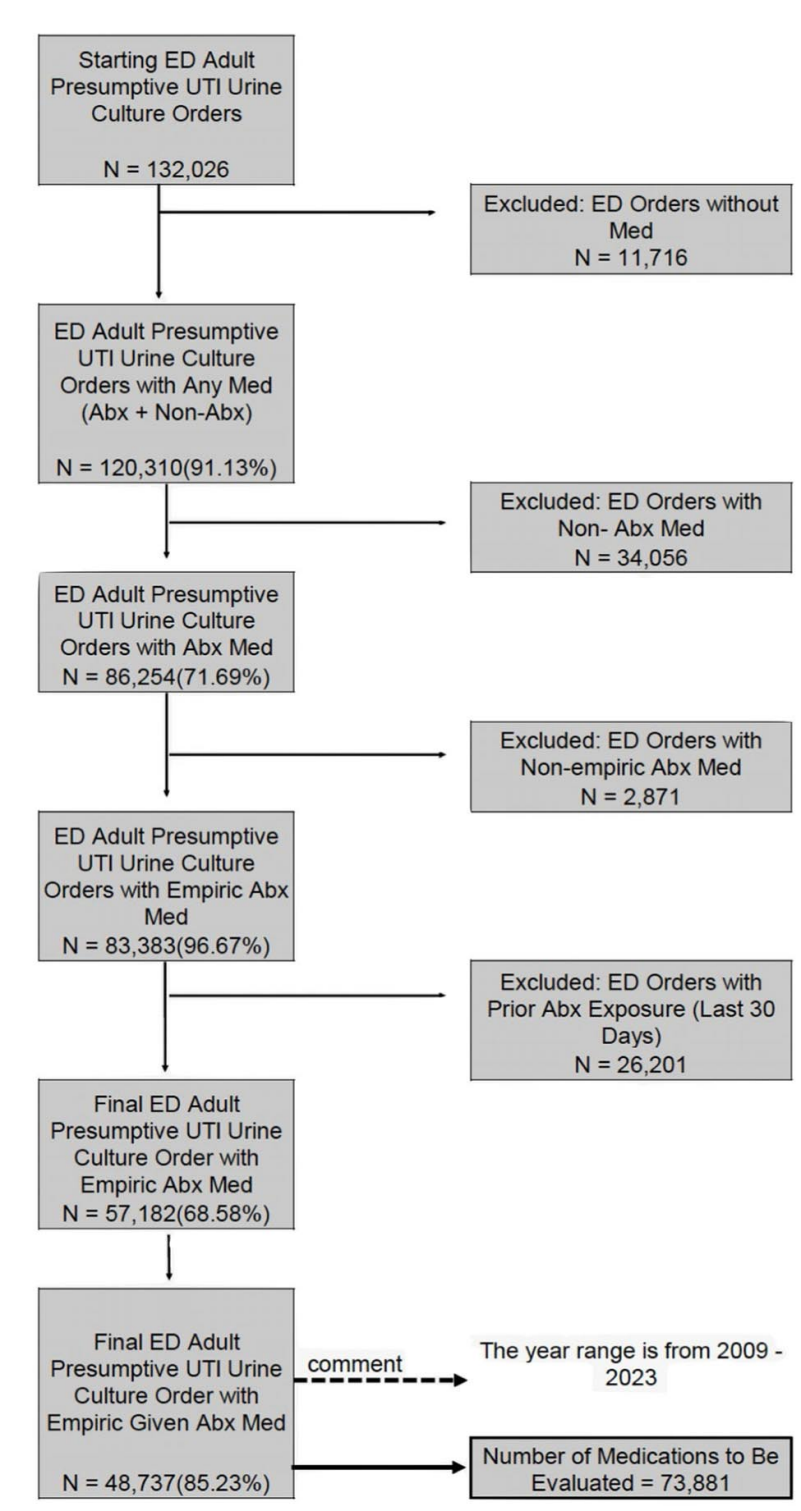
Cohort Construction Flowchart for Adult Emergency Department (ED) Urinary Tract Infection Cases This flow diagram illustrates the cohort generation process for adult ED patients with presumed urinary tract infection (UTI), based on urine culture orders and empiric antibiotic prescriptions. Starting from all urine culture orders, sequential filters were applied to isolate cases from the ED with associated empiric antibiotic treatment, while excluding those with recent antibiotic exposure (within 30 days) or non-relevant encounters. This figure provides a visual example of inclusion and exclusion criteria, culminating in the final analytic sample used in the appropriateness analysis. The same logic was applied to generate the pediatric ED and adult outpatient cohorts (not shown). As a proof-of-concept study using a large real-world dataset, limitations include potential misclassification, missing data, and variability in documentation or data completeness across care settings.

**Figure 2:**
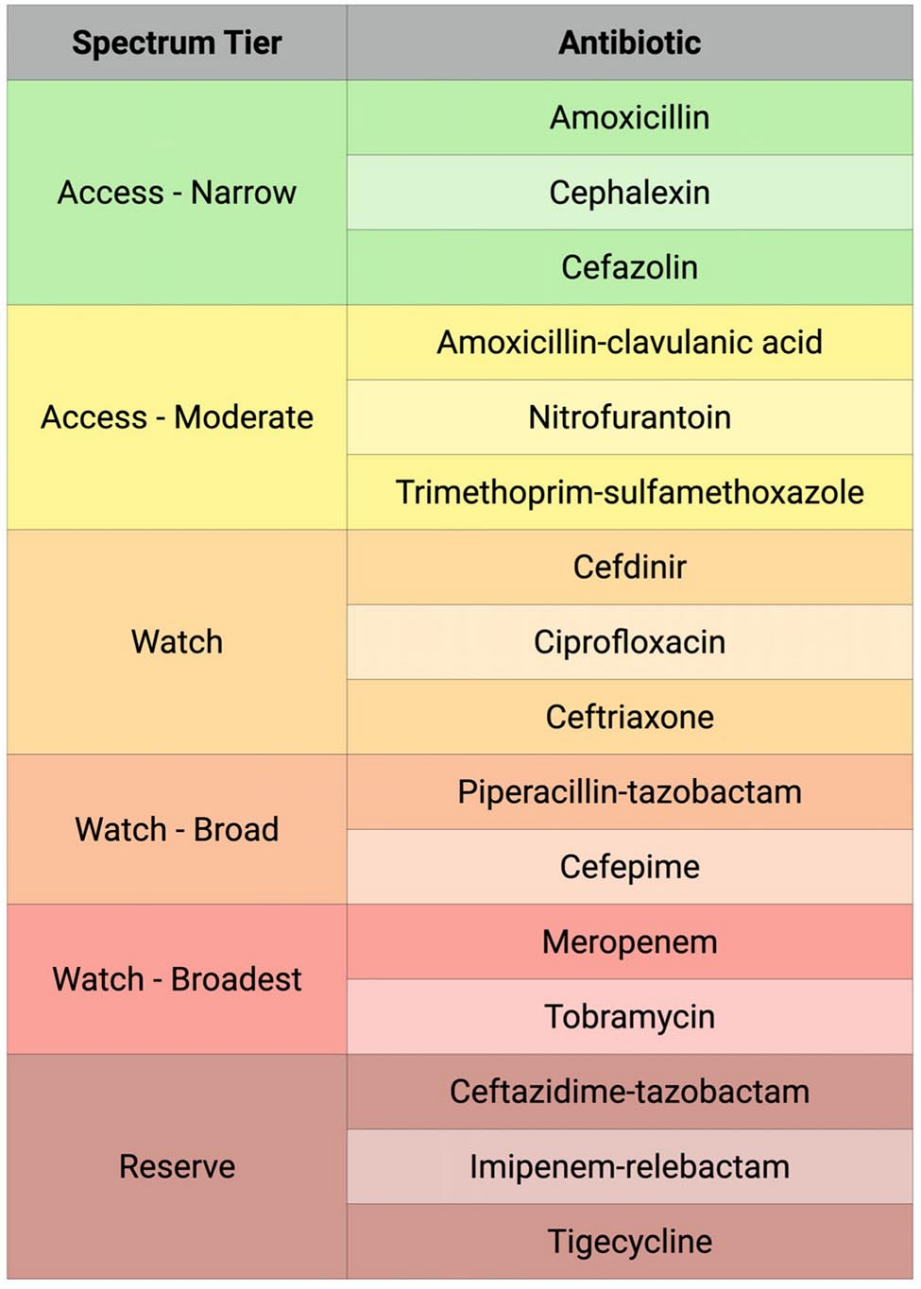
Antibiotic Spectrum Tiering Used for Appropriateness Classification in the DOOR MAT Framework This figure presents selected examples of how empiric antibiotics were categorized into six hierarchical spectrum tiers, adapted from the World Health Organization’s AWaRE classification. AWaRE groups antibiotics into three categories to guide stewardship prioritization: Access (first-line agents with low resistance potential), Watch (higher resistance potential), and Reserve (last-resort agents for multidrug-resistant infections). These categories were expanded to improve granularity for spectrum-based assessment. Narrow-spectrum agents appear in lower tiers, while broader-spectrum and last-resort agents occupy higher tiers. This new tiering system, developed and validated by an infectious diseases physician, served as the basis for evaluating whether a prescribed agent was optimal, broader than necessary (over-treatment), or lacked adequate activity (under-treatment) relative to culture and antimicrobial susceptibility testing (AST) data. The tiers were used in conjunction with SQL-based cohort construction and join logic to apply the DOOR MAT (Desirability of Outcome Ranking for the Management of Antimicrobial Therapy) framework. This is not a comprehensive list; antibiotics shown here are representative examples from the full tiering system.

**Methods:**

We used the ARMD EHR dataset to identify presumptive UTI cases based on urine culture orders and empiric antibiotics, excluding patients with antibiotic exposure in the prior 30 days. Cohorts were stratified by care setting (Figure 1). Antibiotics were categorized into six spectrum tiers adapted from WHO AWaRE and validated by an infectious diseases physician (Figure 2). Using the DOOR MAT framework, we applied SQL logic to compare empiric therapy with culture and antimicrobial susceptibility testing (AST) results, classifying each case as optimal, over-treatment, under-treatment, unnecessary, or not assessable. All unique antibiotics and organisms were retained for full assessment.

**Figure 3:**
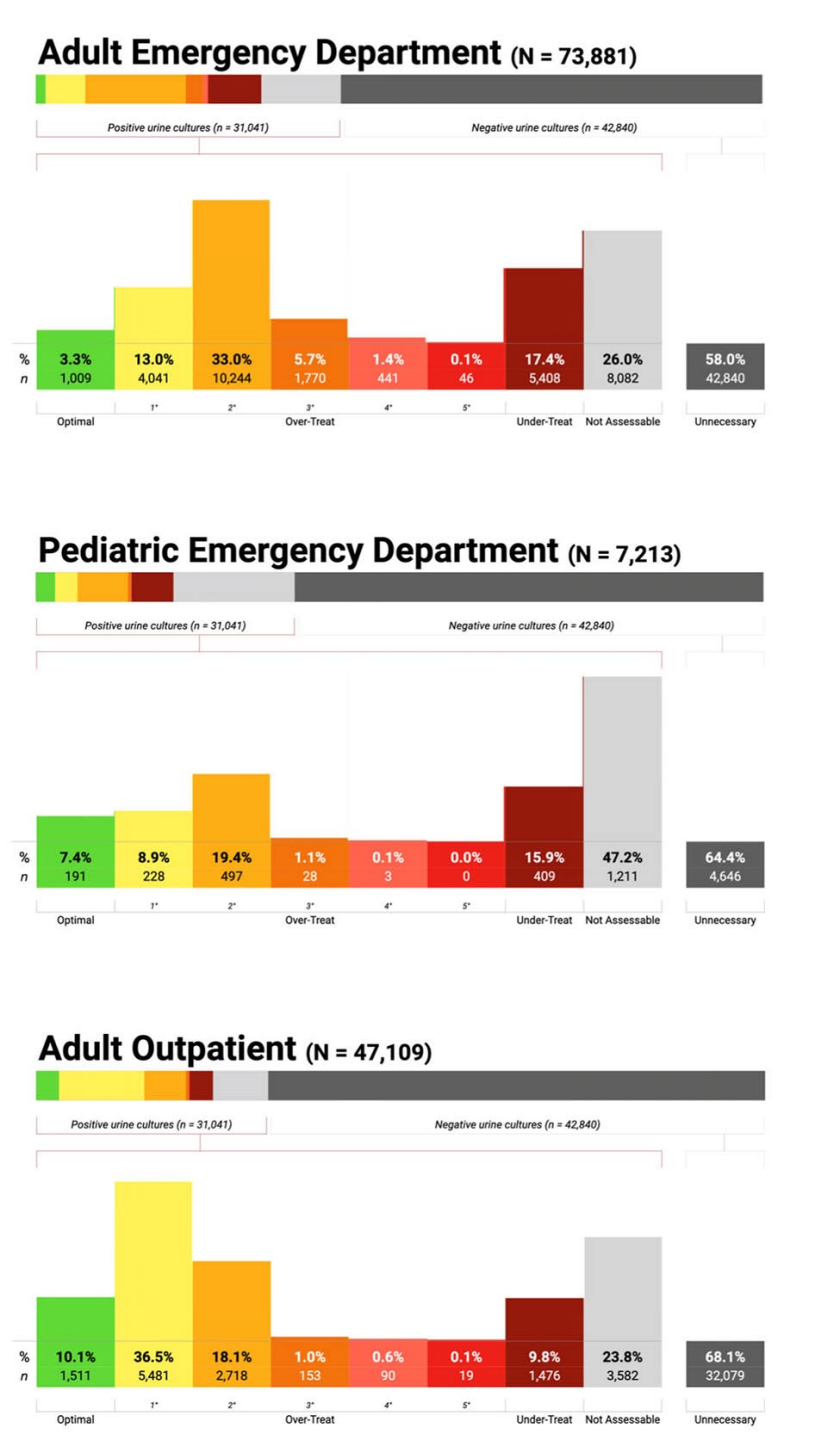
Appropriateness of Empiric Antibiotic Prescribing for Urinary Tract Infections Across Care Settings This figure displays a spectrum-based histogram of empiric antibiotic appropriateness for culture-positive urinary tract infection (UTI) cases across adult ED, pediatric ED, and adult outpatient settings. Prescriptions are classified as optimal (green), indicating the narrowest agent with full in vitro activity based on antimicrobial susceptibility testing (AST) or implied susceptibility; over-treatment (yellow to red gradient), where the agent was active but broader than necessary, with color intensity reflecting the number of spectrum tiers above the optimal choice; under-treatment (dark red), where the agent lacked activity against the cultured organism(s); and not assessable (gray), where AST was unavailable and no intrinsic resistance or predictable susceptibility could be inferred. The histogram includes only culture-positive cases to maintain interpretability. The percentage and count of unnecessary prescriptions (defined as empiric antibiotics given for negative cultures) are shown separately to the right, as inclusion in the main histogram would distort the visual scale. This color-coded format enables intuitive assessment of antibiotic use across departments, where green reflects appropriate prescribing and red indicates increasingly inappropriate use.

**Figure 4:**
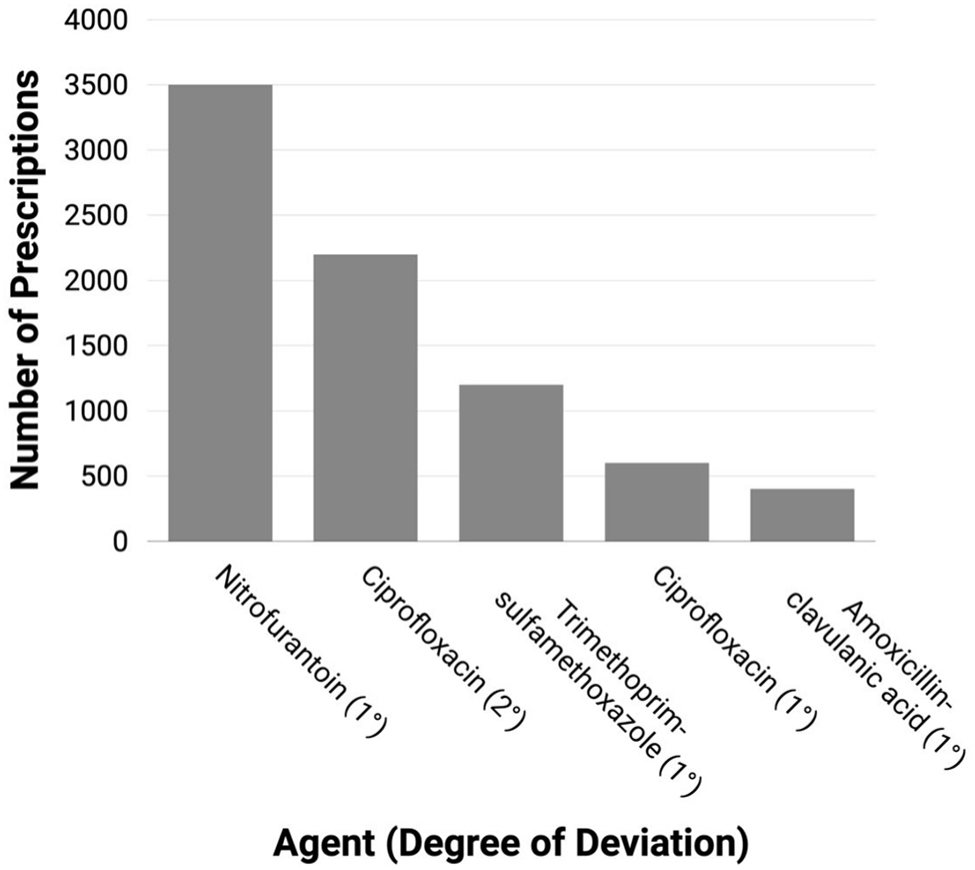
Spectrum Deviation of Commonly Over-Treated Antibiotics in the Adult Outpatient Cohort This bar chart displays the five most frequently prescribed empiric antibiotics in the adult outpatient cohort that were classified as over-treatment based on final urine culture and antimicrobial susceptibility testing (AST) results. Each antibiotic is labeled on the x-axis with the number of spectrum tier deviations above the optimal agent, based on the adapted WHO AWaRE classification system. For example, nitrofurantoin was the most commonly prescribed agent but typically deviated by only one tier from the optimal choice, whereas ciprofloxacin was two tiers broader than necessary in most cases, where an agent such as amoxicillin (lowest tier) would have provided adequate coverage. This figure illustrates how over-treatment varies not only by drug selection but also by degree of unnecessary spectrum, providing insight into stewardship opportunities beyond binary classification alone.

**Results:**

Of 73,881 adult ED prescriptions, 58.0% were unnecessary. Among culture-positive cases, 3.3% were optimal, 53.3% over-treated, 17.4% under-treated, and 26.0% lacked AST. In 7,213 pediatric ED cases, 64.4% were unnecessary; among positives, 7.4% were optimal, 29.5% over-treated, and 47.2% lacked AST. Among 47,109 adult outpatient prescriptions, 68.1% were unnecessary; among positives, 10.1% were optimal, 56.3% over-treated, and 23.8% lacked AST (Figure 3). Nitrofurantoin and ciprofloxacin were the most overused agents in the outpatient setting (Figure 4).

**Conclusion:**

This structured, SQL-based framework enables standardized assessment of empiric antibiotic appropriateness using only EHR data. By determining appropriateness along a spectrum relative to culture and susceptibility results, it offers a scalable alternative to manual audit or rule-based alerts. These reproducible measures can support real-time and longitudinal stewardship, particularly in high-volume or outpatient settings.

**Disclosures:**

Hayden T. Schwenk, MD, MPH, Bristol Myers Squibb: Stocks/Bonds (Public Company)|Karius, Inc.: Consultant, Medical Affairs Jonathan H. Chen, MD, PhD, Reaction Explorer: Ownership Interest

